# Assessment of apoptosis in the native vein used for hemodialysis access

**DOI:** 10.3325/cmj.2016.57.540

**Published:** 2016-12

**Authors:** Laura Leci-Tahiri, Božo Krušlin, Majda Vučić, Zdenko Sonicki, Ivo Lovričević

**Affiliations:** 1 Clinic of Vascular Surgery, University Clinical Center of Kosovo, Prishtina, Kosovo; 2 “Ljudevit Jurak” Department of Pathology, University Hospital Center “Sestre milosrdnice,” Zagreb, Croatia; 3 University of Zagreb School of Medicine, Zagreb, Croatia; 4 Department of Medical Statistics, Epidemiology, and Medical Informatics, Andrija Štampar School of Public Health, University of Zagreb School of Medicine, Zagreb, Croatia; 5 Department of Vascular Surgery, University Hospital Center “Sestre milosrdnice,” Zagreb, Croatia

## Abstract

**Aim:**

To determine whether apoptosis is more common in previously punctured native veins than in non-punctured native veins among patients who undergo surgical creation of arteriovenous fistula (AVF) for dialysis access.

**Methods:**

Cephalic vein specimens were obtained from January 1, 2013 to December 31, 2014 from 60 patients, 30 with previously punctured native veins and 30 with non-punctured native veins. Before AVF placement, a 1-cm vein segment was excised from distal part of the vein for histological, histochemical, and immunohistochemical analysis. Vein specimens were divided into two portions along the longitudinal axis and stained with hematoxylin and eosin for routine histological evaluation. Immunohistochemical analysis was used to localize Bax, p53, caspase 3, and Bcl-2 expression.

**Results:**

The group with previously punctured veins showed significantly increased caspase 3 (*P* < 0.001, two-sided Fisher`s Exact Test) and Bax expression (*P* = 0.002, two-sided Fisher`s Exact Test) and significantly decreased Bcl-2 expression (*P* < 0.001, two-sided Fisher`s Exact Test) compared with the control group. There were no significant differences between the groups in p53 expression (χ^2^ = 0.071, df = 1, *P* = 0.791). Fistula failure was significantly more common in the study group (26.7% vs 6.7%, χ^2^ = 4.32, df = 1, *P* = 0.038).

**Conclusion:**

Our study indicates a possible role of venipuncture in apoptosis development and a possible role of apoptosis in fistula failure, but we do not have sufficient evidence to conclude that it represents its main cause.

The past two decades have seen a significant increase in the number of patients with end-stage renal disease requiring hemodialysis and in the mortality rates of hemodialysis patients. A common problem in hemodialysis patients is arteriovenous fistula (AVF) failure, a major cause of morbidity and hospitalization (1,2). Early native AVF failure (juxta-anastomotic stenosis) has a complex pathogenesis, and predisposing factors include a small artery (<1.5 to 2 mm) and a small vein (<2.0 to 2.5 mm), surgical manipulation and less-than-ideal technique, previous punctures, development of accessory veins that re-direct blood flow away from the primary venous drainage channel, hemodynamic stressors, and a genetic predisposition to vasoconstriction and neointimal hyperplasia after endothelial and smooth muscle injury (3-7).

Abnormalities in the apoptotic cell death control contribute to a variety of cardiovascular diseases such as atherosclerosis, aneurysm formation, ischemic cardiomyopathies, infarction, and varicose veins (8-10). Several studies have dealt with apoptosis in the varicose veins of the lower extremities (11,12), but there has been no research on the upper extremities veins. The only way to achieve this was to study the veins used for hemodialysis access. Apoptosis has a complex pathway, and various mediators regulating apoptosis and cell proliferation have been evaluated so far. It was found that cells with a mutated p53 gene cannot control genomic integrity and tend to escape from apoptosis (9). Bcl-2 family proteins, including pro- and antiapoptotic members, participate in the p53 apoptotic pathway, and the equilibrium between these positive and negative regulatory proteins is essential for creating susceptibility to apoptosis (11,12).

The aim of the study was to evaluate apoptosis presence and extent in previously punctured and non-punctured native veins used to create AVF in hemodialysis patients. We also aimed to assess the number of patients with AVF failure and assess its association with apoptosis.

## Materials and methods

### Patients

Cephalic vein specimens were obtained from 60 patients with chronic renal disease (CRD) at the University Clinical Center of Kosovo from January 1, 2013 to December 31, 2014. The number of patients was determined on the basis of a similar study (13). The study group included patients with a previously punctured vein used to create AVF (n = 30) and the control group included patients with no previously punctured vein (n = 30). The indication for AVF creation was established according to the international recommendations (14-16).

All included patients had a competent deep venous system without a history of thrombotic episodes and systolic blood pressure higher than 100 mm Hg. The study did not include patients in whom AVF was not developed to sustain dialysis or who were thrombosed before the first successful cannulation, patients in whom AVF was created with graft prosthesis, and patients with stenosis and thrombosis of the draining or central veins.

For each patient we collected data on age, sex, time of AVF creation, duration of CRD and dialysis, anatomic position of AVF, type of AVF, Doppler sonographic data before the creation of AVF, presence of comorbid conditions (diabetes mellitus, coagulation disorder, malignancy, coronary artery disease, cerebrovascular disease, peripheral vascular disease, use of angiotensin-converting-enzyme inhibitor inhibitors, statins, calcium antagonist, coumarin, platelet aggregation inhibitor, prior central catheter placement, hepatitis B surface antigen, hepatitis C virus, human immunodeficiency virus, history of intravenous drug abuse, smoking). The history of venipuncture was obtained from the patient or patient’s family member. Preoperative sonographic mapping (Siemens Acuson X300 system [Erlangen, Germany]; 7 to 15 MHz transducer) was performed by a vascular surgeon.

### Preoperative evaluation

The veins were sequentially evaluated along the arm for diameter, patency, and depth. The exclusion criteria were stenosis and thrombosis of the draining or central veins. The appropriateness of the arteries and veins to create the AVF was based on the level of arterial atherosclerosis, blood flow, and the presence of thrombosis, and was assessed as excellent, good, or sufficient (Table 1).

**Table 1:** The level of appropriateness of the arteries and veins to create the arteriovenous fistula

Arteries	Internal diameter	Wall	Flow
**excellent**	**minimum of 2 mm**	**no atheroma**	**good**
**good**	minimum of 2 mm	stiff artery wall with no atheroma	average to good
**sufficient**	minimum of 2 mm	hard and fragile artery wall with atheroma	average
**Veins**			
**excellent**	minimum of 2 mm	without any clots and thrombosis, completely open proximal	good flow, dilates and fills well with pressure on the proximal part of the vein
**good**	minimum of 2 mm	without proximal obstruction, persisted proximal stenosis, which could be removable with dilatators	the vein is appropriately filled with blood after release of proximal pressure
**sufficient**	minimum of 2 mm	stiff wall, stenosis, or obstruction of the proximal part, dilatators of maximum size of 2 Fresenius could pass through	low flow rate, proximal part without appropriate blood and adequate dilatation

### Surgical technique

Surgical procedure was performed on an outpatient basis (local anesthesia). The AVFs were created at the non-dominant extremity. The patient was supine with the examined arm comfortably extended approximately 60° from the chest. AVFs were created in the usual manner (radial artery-cephalic vein, brachial artery-cephalic vein, termino-lateral anastomosis [end-to-side] using 6/0 or 7/0 polypropylene suture). The cephalic vein was excised. Intraoperatively, before creating AVF, a 1-cm long vein segment was excised from its distal part for histological and immunohistochemical analysis.

### Specimens

The vessel samples were irrigated and dilated with heparin-saline solution. AVF maturation was sonographically observed during 4-6 weeks depending on the patient's general condition. AVF was considered to be sufficiently matured when blood flow reached 500 mL/min and vessels' diameter was 4 mm (3,14,17,18). Fistula failure was defined as late failure, ie, an inability to use the matured AVF after at least 3 months of normal usage. Length of follow up to failure was one year after the first fistula puncture. Patients were followed up on 3, 6, 9, and 12 months, and AVF failure was noted. Hemodialysis was performed using the standard procedure; each procedure lasted 4 hours, 3 times weekly using biocompatible polysulphone hemodialysis membranes (Fresenius, Grove City, OH, USA) (15).

### Tissue preparation

The collected specimens were fixed in 10% buffered solution containing 4% formaldehyde for 24 hours and embedded in paraffin for conventional histology, histochemistry, and immunohistochemistry.

### Histology and histochemical staining

Specimens for histological evaluation were stained with hematoxylin and eosin (H&E). Results were obtained by two independent investigators who were blinded to the patient’s clinical findings. Mallory, Gomori, and Van Gieson's elastotic staining were used to evaluate morphological changes in the veins.

### Immunohistochemistry

For immunohistochemical analysis of apoptosis we selected the most commonly used antibody panel: p53 mouse monoclonal antibody in a 1:50 dilution; Bcl-2 mouse monoclonal antibody in a 1:50 dilution; caspase 3 mouse monoclonal antibody in a 1:50 dilution; and Bax rabbit polyclonal antibody in a 1:500 dilution (all from DAKO, Glostrup, Denmark) (13,18).

Streptavidin-biotin-peroxidase method was performed according to the manufacturer’s instructions (DAKO). Biotin-conjugated secondary antibody was applied in a 1:200 dilution for 1 hour at room temperature, followed by a 30-minute incubation in Strept-AB complex for color development and counterstaining with 3,3′-diaminobenzidine tetrahydrochloride and hematoxylin. The protein expression was evaluated using a semiquantitative assessment of positive cells by Filis et al (13). Scoring was as follows: no staining, minimum staining (1%-3%), moderate staining (>3%-50%), and maximum staining (>50%-100%). Cells that stained positive (cytoplasmic and nuclear staining) for the examined antibodies in the intima, media, and adventitia were counted at 400 × magnification and quantified in 10 random fields per section. We used the tonsil as positive control for Bcl-2, caspase 3, and Bax, and breast tissue for p53. Negative control was obtained by omitting primary antibody.

Ethical approval was received from the University Clinical Center of Kosovo, Prishtina, University Hospital Center “Sestre milosrdnice,” Zagreb, and University of Zagreb School of Medicine. Informed consent was obtained from all participants.

### Statistical analysis

Chi square test or Fisher Freeman Halton test were used to test the differences in the proportions of qualitative variables between the groups. Normality of quantitative variable distributions was tested by Kolmogorov-Smirnov test. Mann-Whitney U was used to test the differences between quantitative variables that were not normally distributed. *P* < 0.05 was considered significant. For statistical analysis we used Dell Statistica, version 13 (Round Rock, TX, USA).

## Results

According to preoperative physical examination and noninvasive imaging findings, 60 patients on maintenance hemodialysis with an AVF as vascular access were found to be appropriate candidates for this study (Table 2).

**Table 2:** Characteristics of patients with (study group) and without previously punctured native veins (control group)

	Study group (N = 30)	Control group (N = 30)	*P*
**Age (years), median/interquartile range**	63.5/25	63/20.25	0.695**^†^**
**Sex (male/female)**	18/12	20/10	0.592*
**No dialysis**	11 (36.7)	10 (33.3)	0.787*
**Central venous catheter**			0.713*
internal jugular vein	6 (20.0)	9 (30.0)	
femoral vein	11 (36.7)	8 (26.7)	
subclavian vein	2 (6.7)	3 (10.0)	
**Serology**			
Hepatitis B surface antigen positive	2 (6.7)	2 (6.7)	1.000*
Hepatitis C virus positive	1 (3.3)	2 (6.7)	1.000*
Human immunodeficiency virus positive	0 (0)	0 (0)	
**Comorbidities**			
hypertension	26 (86.7)	21 (70.0)	0.117*
pulmonary disease	3 (10.0)	3 (10.0)	1.000*
hematologic disease	19 (63.3)	17 (56.7)	0.598*
diabetes	11 (36.7)	11 (36.7)	1.000*
peripheral vascular disease	2 (6.7)	2 (6.7)	1.000*
cerebrovascular disease	4 (13.3)	0 (0)	0.112*
rheumatologic disease	2 (6.7)	1 (3.3)	1.000*
cancer	2 (6.7)	5 (16.7)	0.424*
**Choice of access site**			0.254*
- non dominant extremity	24 (80.0)	28 (93.3)	
- dominant extremity	6 (20.0)	2 (6.7)	
**Type of fistula**			0.892*
- radial-cephalic fistula sin	15 (50.0)	15 (50.0)	
- brachial-cephalic sin	10 (33.3)	12 (40.0)	
- radial-cephalic fistula dex	3 (10.0)	2 (6.7)	
- brachial-cephalic dex	2 (6.7)	1 (3.3)	

*Fischer’s exact test.

**†**Mann-Whitney U test.

### Histology and histochemical staining

**Figure 1:**
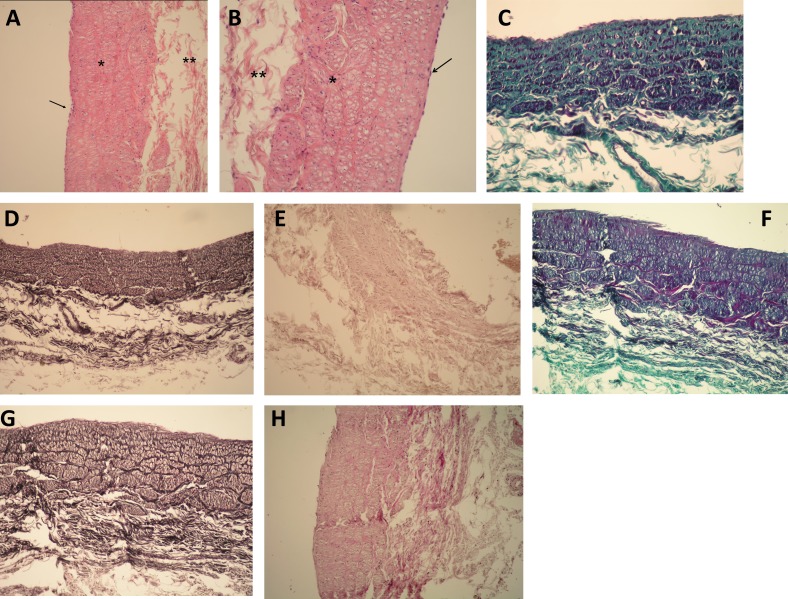
(**A**) Control vein, hematoxylin and eosin staining, ×100. Hyphen indicates the intima (endothelium); one asterisk indicates the media; two asterisks indicate the adventitia. (**B**) Punctured vein, hematoxylin and eosin staining, ×200. Hyphen indicates the intima (endothelium); one asterisk indicates the media; two asterisks indicate the adventitia. (**C,D,E**) Control vein. (**C**) Mallory trichrome staining; (**D**) Gomori’s staining; (**E**) Van Gieson’s staining. (**F,G,H**) Punctured vein. (**F**) Mallory trichrome staining; (**G**) Gomori’s staining; (**H**) Van Gieson’s staining.

Routine H&E staining showed histological appearance of punctured and non-punctured vein specimens. Signs of apoptosis were visible in the form of pyknotic bodies. Mallory and Gomori’s histochemical staining demonstrated a loss of elongated morphology of punctured veins and slightly increased collagen matrix, while Van Gieson’s elastic staining showed degradation of elastic fibers compared with non-punctured veins (Figure 1).

### Expression of apoptotic and antiapoptotic markers (IHC)

**Figure 2:**
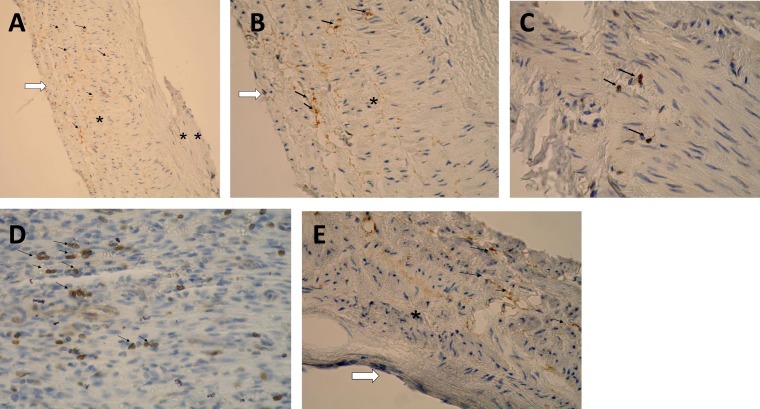
(**A**) Positive cytoplasmatic immunohistochemical staining of Bax in the media and intima of the punctured vein, ×200. White hyphen indicates the intima; one asterisk indicates the media; two asterisks indicate the adventitia; small black hyphens indicate positive cytoplasmatic staining. (**B**) Positive cytoplasmatic immunohistochemical staining of caspase 3 in the media and intima of the punctured vein, ×400. White hyphen indicates the intima; one asterisk indicates the media, small black hyphens indicate positive cytoplasmatic staining. (**C**) Positive nuclear immunohistochemical staining of p53 in the media of the punctured vein, ×400. Small black hyphens indicate positive nuclear staining. (**D**) Nuclear immunohistochemical positivity of Bcl-2 in the media of the control vein, ×400. Positive reaction, visible as dark staining in the nucleus (black arrows). (**E**) Positive cytoplasmatic immunohistochemical staining of caspase 3 in the media and intima of the control vein, ×400. White hyphen indicates the intima; black hyphen indicates positive cytoplasmatic staining; asterisk indicates the media.

In the study group, p53 showed no or minimal expression, Bcl-2 showed minimal and moderate expression, caspase 3 showed minimal, moderate, and maximal expression, while Bax showed no or minimal and moderate expression (Figure 2A-C and Table 3). In the control group, p53 showed no or minimal expression, Bcl-2 showed minimal, moderate, and maximal expression, caspase 3 showed no or minimal expression, and Bax showed no or minimal expression (Figure 2D, E and Table 3).

**Table 3:** Comparison of apoptotic and antiapoptotic markers expression between patients with (study group) and without previously punctured native veins (control group)

	Study group	Control group	*P*
**p53**			0.791*
no staining	18	19	
minimum staining^†^	12	11	
moderate staining	0	0	
maximum staining	0	0	
**Bcl-2**			<0.001*
no staining	0	0	
minimum staining	20	3	
moderate staining	10	20	
maximum staining	0	7	
**Caspase 3**			<0.001*
no staining	0	19	
minimum staining	6	11	
moderate staining	18	0	
maximum staining	6	0	
**Bax**			<0.002*
no staining	11	23	
minimum staining	14	7	
moderate staining	5	0	
maximum staining	0	0	

*Fischer’s exact test.

†Minimum staining (1%-3% positive cells), moderate staining (>3%-50% positive cells), maximum staining (>50%-100% positive cells).

The study group showed significantly increased caspase 3 and Bax expression (*P* < 0.001, and *P* = 0.002, two-sided Fisher`s Exact Test, respectively) and significantly decreased Bcl-2 expression compared with the control group (*P* < 0.001, two-sided Fisher`s Exact Test). There were no significant differences between the groups in p53 expression (χ^2^ = 0.071, df = 1, *P* = 0.791) (Table 3). Fistula failure was significantly more common in the study group (26.7% vs 6.7%, χ^2^ = 4.32, df = 1, *P* = 0.038) (Table 4).

**Table 4:** Fistula failure in patients with (study group) and without previously punctured native veins (control group)

	Study group	Control group	Total
Fistula failure, % within group	8 (26.7)	2 (6.7)	10 (16.7)

## Discussion

This study found increased caspase 3 and Bax expression and decreased Bcl-2 expression in the study group compared with the control group and no significant differences in p53 expression between the groups. No similar studies have been published so far, but there are studies dealing with apoptosis in different vascular lesions, especially atherosclerosis and varicose veins. Specimens retrieved from patients with AVF restenosis more frequently contained apoptosis foci than specimens of primary atherosclerotic lesions (19). The adventitia of varicose vein specimens showed lack of immunopositivity for Bax, while atherosclerotic lesions of primary and restenotic types showed a lack of immunopositivity for Bcl-2 (13,19-22). cfDNA released from blood cells by ongoing apoptosis was abundantly present in the plasma of hemodialysis patients (23). Their plasma mimicked the capacity of cfDNA to induce IL-6 in human monocytes, indicating that this process may contribute to the proinflammatory environment observed in hemodialysis patients (23).

Filis et al (13) and Ascher et al (20) explained the lack of Bcl-2 expression in varicose veins by the lack of specificity of the antibody used to cell types in the vein tissue. Filis et al (13) also observed a significant difference in caspase-3 immunopositivity between varicose vein group and control group, suggesting an active apoptotic state in varicose veins. We observed a significant difference in caspase-3 expression between study and control group, suggesting an active apoptotic state in previously punctured veins used for hemodialysis access. Our results are comparable with the results of previous studies that found no completely specific apoptotic marker for apoptotic cells, requiring the use of a combination of techniques (13,24-27).

Filis et al (13) found p53 expression only in the tributary and distal great saphenous vein (GSV) of the control group consisting of patients with healthy GSV used for by-pass grafting in open heart surgery. Urbanek et al (28) also found increased p53 expression in the distal GSV of young patients. There is evidence that surgical trauma and modified hemodynamics are associated with endothelial and smooth muscle cell damage. Langer et al (29) presumed that the uremic environment rather than indirect effects exacerbated neointimal hyperplasia and calcification within the AVF. In the literature there is a discrepancy concerning the Bcl-2 expression in human medial smooth muscle cells (30,31). Hayakawa et al (32) reported that Bax protein expression increased with the progression of atherosclerosis, but they did not observe Bcl-2 expression. They found that the only parameter affecting the maturation time of brachio-basilic fistula was vein diameter. AV fistulas using basilica veins with diameters larger than 3 mm were shown as suitable for hemodialysis in the short term (6,15,30).

Our study found fistula failure to be more common in the study group. Smith et al (33) studied samples of the brachial vein used to create AVFs in 15 hemodialysis patients, showing significantly greater intimal and medial widths in patients who had been treated with hemodialysis less than 6 months than in those who had been treated for more than 6 months. Moreover, the results showed that the present recommendations for a minimum diameter (2 mm) for a radial artery and a cephalic vein were sufficient for adequate AVF creation at the wrist. To compare previously published data with the results of our study we strictly followed the mentioned recommendations for AVF creation in all included patients.

A limitation of our study was that there is no specific marker that solely detects apoptotic cells so that the application of different techniques would improve the quality of our results. Also, the analysis was performed on a relatively small number of patients.

In conclusion, our study indicates that previously punctured native veins used for hemodialysis exhibit increased apoptotic activity (caspase 3, Bax) compared with non-punctured veins, which suggests an increased risk of AVF failure in patients with previously punctured veins. Further analysis on a larger number of specimens is recommended.

## References

[1] Nissenson AR (2014). Improving outcomes for ESRD patients: shifting the quality paradigm.. Clin J Am Soc Nephrol..

[2] Nikeghbalian S, Bananzadeh A, Yarmohammadi H (2006). Difficult vascular access in patients with end-stage renal failure.. Transplant Proc.

[3] Pantea S, Bengulescu I (2014). Smooth loop arterio-venous fistula.. Chirurgia (Bucur).

[4] Gelabert HA, Freischlag JA. Hemodialysis access. In: Rutherford R. Vascular surgery. Philadelphia: W.B. Saunders Co; 2000. p. 1466-76.

[5] Ferrari G, Talassi E, Baraldi C, Baruffaldi M, Tarchini R, Galli E (2005). A good vascular access allows an effective treatment.. G Ital Nefrol.

[6] Konner K, Nonnast-Daniel B, Ritz E (2003). The arteriovenous fistula.. J Am Soc Nephrol.

[7] Allon M, Robbin ML (2002). Increasing arteriovenous fistulas in hemodialysis patients: problems and solutions.. Kidney Int.

[8] Clarke M, Bennett M, Littlewood T (2007). Cell death in the cardiovascular system.. Heart.

[9] Lee Y, Gustafsson AB (2009). Role of apoptosis in cardiovascular disease.. Apoptosis.

[10] Ducasse E, Giannakakis K, Speziale F, Midy D, Sbarigia E, Baste JC (2008). Association of primary varicose veins with dysregulated vein wall apoptosis.. Eur J Vasc Endovasc Surg.

[11] Ascher E, Jacob T, Hingorani A, Gunduz Y, Mazzariol F, Kallakuri S (2000). Programmed cell death (apoptosis) and its role in the pathogenesis of lower extremity varicose veins.. Ann Vasc Surg.

[12] Walsh K, Smith RC, Kim HS (2000). Vascular cell apoptosis in remodeling, restenosis and plaque rupture.. Circ Res.

[13] Filis K, Kavantzasb N, Isopoulosa T, Antonakis P, Siagalas P, Vavouranakis E (2011). Increased vein wall apoptosis in varicose vein disease is related to venous hypertension.. Eur J Vasc Endovasc Surg.

[14] Davidson I. The end stage renal disease patient as related to dialysis. In: Access for dialysis: surgical and radiologic procedures, 2nd ed. Austin: Landes Bioscience; 2002. p. 1-10.

[15] Denker B, Chertow G, Owen W. Hemodialysis. In: Brenner B. The kidney. Philadelphia: W.B. Saunders Co; 2000. p. 2373-457.

[16] Szczech LA, Harmon W, Hostetter Th, Klotman P, Powe N, Sedor J (2009). World kidney day 2009: Problems and challenges in the emerging epidemic of kidney disease.. J Am Soc Nephrol.

[17] Atamaniuk J, Kopecky C, Skoupy S, Saemann MD, Weichhart T (2012). Apoptotic cell-free DNA promotes inflammation in haemodialysis patients.. Nephrol Dial Transplant.

[18] Carracedo J, Ramirez R, Madueno JA, Soriano S, Rodrigues-Benota A, Rodrigues M (2002). Cell apoptosis and hemodialysis-induced inflammation.. Kidney Int Suppl.

[19] Isner JM, Kearney M, Bortman S, Passeri J (1995). Apoptosis in human atherosclerosis and restenosis.. Circulation.

[20] Ascher E, Jacob T, Hingorani A, Tsemekhin B, Gunduz Y (2001). Expression of molecular mediators of apoptosis and their role in the pathogenesis of lower-extremity varicose veins.. J Vasc Surg.

[21] Tsujimoto Y (1998). Role of Bcl-2 family proteins in apoptosis: apoptosomes or mitochondria?. Genes Cells.

[22] Ascher E, Hanson JN, Salles-Cunha S, Hingorani A (2003). Lesser saphenous vein thrombophlebitis: its natural history and implications for managment.. Vasc Endovascular Surg.

[23] Atamaniuk J, Kopecky C, Skoupy S, Säemann MD, Weichhart T (2012). Apoptotic cell free DNA promotes inflammation in haemodialysis patients.. Nephrol Dial Transplant.

[24] Kumar V, Abbas AK, Fausto N, Aster JC. Apoptosis. In: Robbins and Cotran. Pathologic basis of disease, 8th ed. Philadelphia: Elsevier Saunders; 2010:25-32.

[25] Sanz AB, Santamaría B, Ruiz-Ortega M, Egido J, Ortiz A (2008). Mechanisms of renal apoptosis in health and disease.. J Am Soc Nephrol.

[26] Hawes D, Shi S, Dabbs D, Taylor C, Cote R. Immunohistochemistry. In: Weidner N, Cote RJ, Suster S, Weiss LM. Modern surgical pathology. Philadelphia: Elsevier Saunders; 2009. p. 48-52.

[27] McIlwain DR, Berger T, Mak TW (2013). Caspase functions in cell death and disease.. Cold Spring Harb Perspect Biol.

[28] Urbanek T, Skop B, Ziaja K, Wilczok T, Wiaderkiewicz R, Palasz A (2004). Sapheno-femoral junction pathology, molecular mechanism of saphenous vein incompetence.. Clin Appl Thromb Hemost.

[29] Langer S, Kokozidou M, Heiss C, Kranz J, Kessler T, Paulus N (2010). Chronic kidney disease aggravates arteriovenous fistula damage in rats.. Kidney Int.

[30] Urbanek T, Skop B, Wiaderkiewicz R, Wilczok T, Zoaja K, Lebda-Wyborny T (2004). Smooth muscle cell apoptosis in primary varicose veins.. Eur J Vasc Endovasc Surg.

[31] Bujan J, Jimenez-Cossio JA, Jurado F, Gimeno MJ, Pascual G, Garcia-Honduvilla N (2000). Evaluation of the smooth muscle cell component and apoptosis in the varicose vein wall.. Histol Histopathol.

[32] Hayakawa Y, Takemura G, Misao J, Kanoh M, Ohno M, Ohashi H (1999). Apoptosis and overexpression of bax protein and bax mRNA in smooth muscle cells within intimal hyperplasia of human radial arteries: analysis with arteriovenous fistulas used for hemodialysis.. Arterioscler Thromb Vasc Biol.

[33] Smith GE, Gohil R, Chetter I (2012). Factors affecting the patency of arteriovenous fistulas for dialysis access.. J Vasc Surg.

